# Supplementary material for: Polymorphisms in Pfkelch13 domains before and after the introduction of artemisinin-based combination therapy in Southwest Nigeria
Source: PLoS One. 2025 Mar 31;20(3):e0316479. doi: 10.1371/journal.pone.0316479 (PMC11957316; doi:10.1371/journal.pone.0316479)

Samples: 13898
Bases: 1177
Average spacing: 12

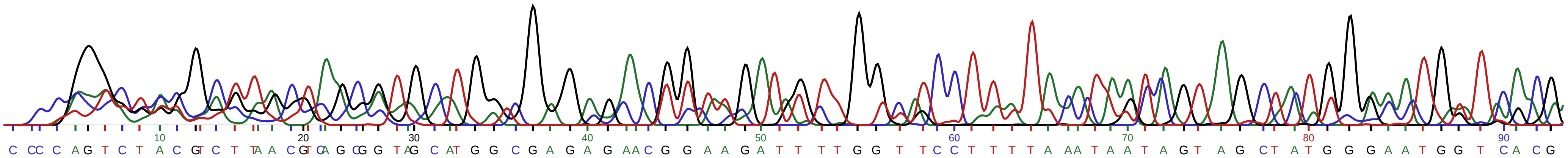

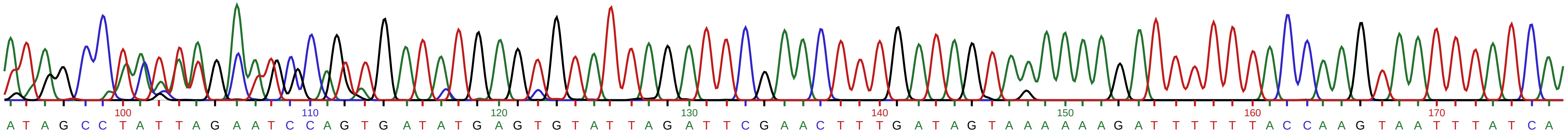

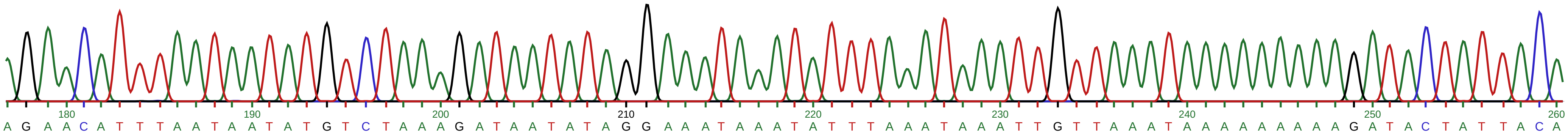

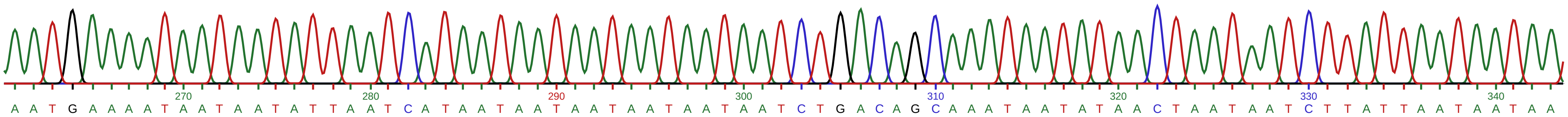

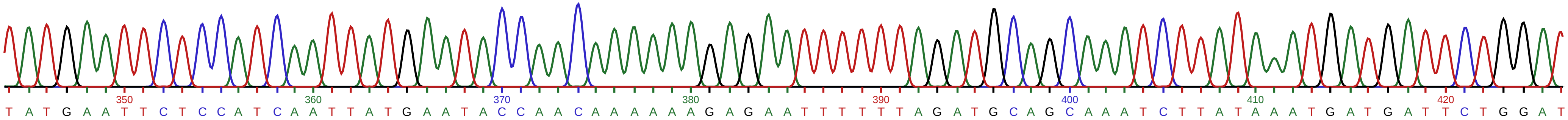

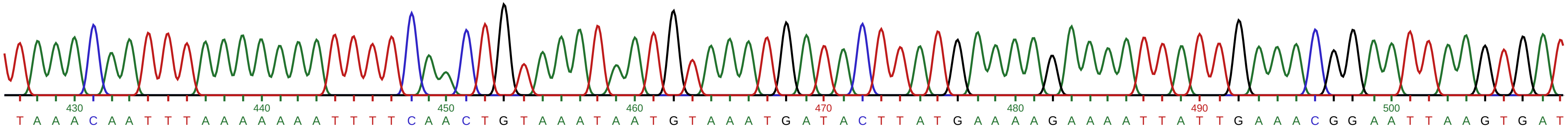

Samples: 13898
Bases: 1177
Average spacing: 12

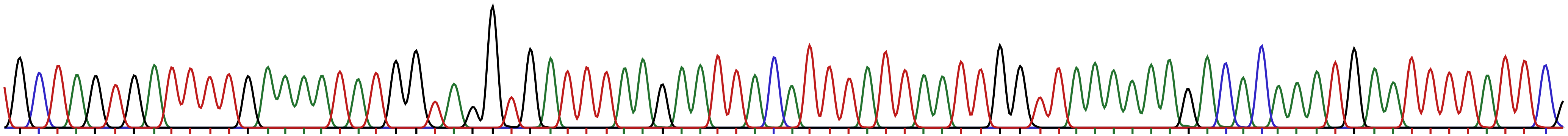

510 520 530 540 550 560 570 580 590

G C T A G T G A T T T T G A A A A T A T G G T A G G T G A T T T A A G A A T T A C A T T T A T T A A T T G G T T A A A A A A G A C A C A A A T G A A T T T T A T T C

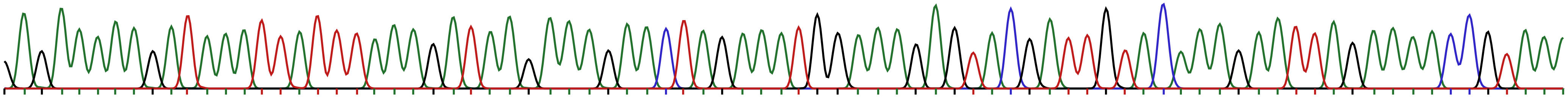

600 610 620 630 640 650 660 670

G A G A A A A A G A T A A A T T A T T T A A A G A T A A G A A A G A A C T A G A A A T G G A A A G A G T A C G A T T G T A C A A A G A A T T A G A A A A C C G T A A A

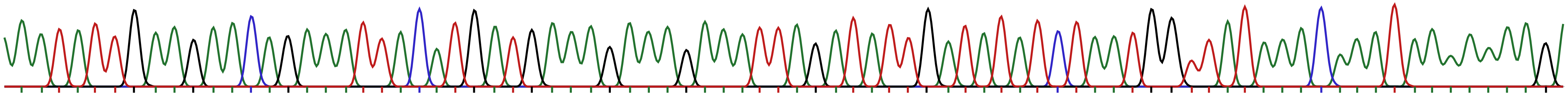

680 690 700 710 720 730 740 750

A A T A T T G A A G A A C A G A A A T T A C A T G A T G A A A G A A A G A A A T T A G A T A T T G A T A T A T C T A A T G G T T A T A A A C A A A T A A A A A A A G

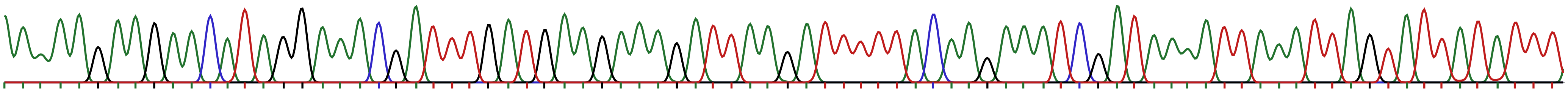

760 770 780 790 800 810 820 830 840

A A A A A G A A G A A C A T A G G A A A C G A T T T G A T G A A G A A A G A T T A A G A T T T T T A C A A G A A A T C G A T A A A A T T A A A T T A G T A T T A T A T T T

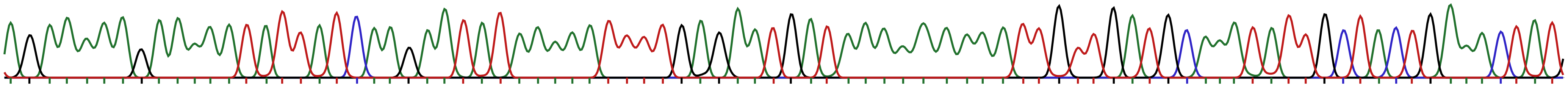

850 860 870 880 890 900 910 920

A G A A A A A G A A A A A T A T T A T C A A G A A T A T A A A A A T T T T G A G A A T G A T A A A A A A A A A A T T G T T G A T G C A A A T A T T G C T A C T G A A A C T A T

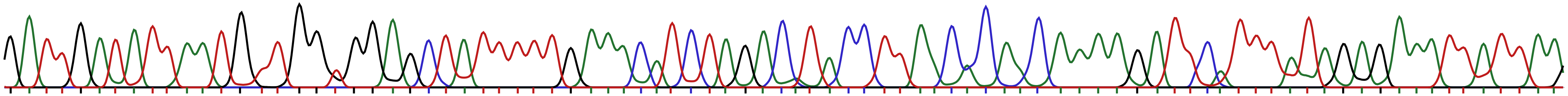

930 940 950 960 970 980 990 1000 1010

G A T T G A T A T T A A T G T T G G T G G A G C T A T T T T T G A A A C A T C T A G A C A T A C C T T A A C A C A A C A A A A G A T T C A T T T A T A G A G A A A T T A T T A A

Samples: 13898
Bases: 1177
Average spacing: 12

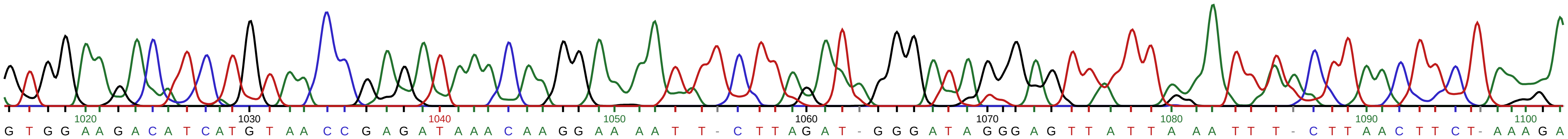

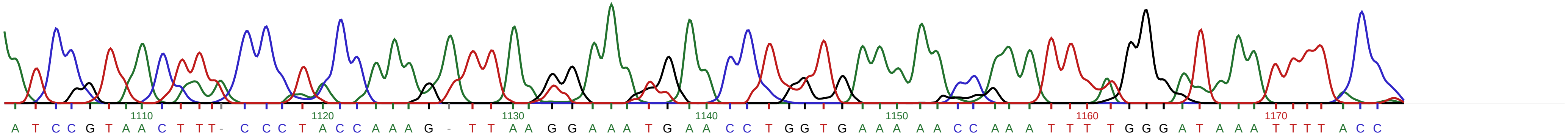

Supplement: Supporting information 2 — (ZIP) [file pone.0316479.s002.zip › 035KN2F_PREMIX_Plate_KELCH1_H04.pdf]
